# Is survival after transanal total mesorectal excision (taTME) worse than that after traditional total mesorectal excision? A retrospective propensity score-adjusted cohort study

**DOI:** 10.1007/s00384-023-04591-7

**Published:** 2024-02-20

**Authors:** Yanic Ammann, Rene Warschkow, Bruno Schmied, Diego De Lorenzi, Christoph Reißfelder, Stephan Bischofberger, Lukas Marti, Walter Brunner

**Affiliations:** 1 Department of General, Visceral, Endocrine and Transplant Surgery, Cantonal Hospital of St. Gallen, Rorschacherstrasse 95, CH-9007 St. Gallen, Switzerland; 2 Department of Surgery, Spital Grabs, Grabs, Switzerland; 3 Department of Surgery, Medical Faculty Mannheim, Universitätsmedizin Mannheim, Heidelberg University, Theodor-Kutzer-Ufer 1-3, 68167 Mannheim, Germany; 4 Department of Surgery, Paracelsus Medical University Salzburg, Salzburg, Austria

**Keywords:** Transanal total mesorectal excision, Laparoscopic total mesorectal excision, Rectal cancer, Survival, Local recurrence

## Abstract

**Purpose:**

Transanal total mesorectal excision (taTME) was developed to provide better vision during resection of the mesorectum. Conflicting results have shown an increase in local recurrence and shorter survival after taTME. This study compared the outcomes of taTME and abdominal (open, laparoscopic, robotic) total mesorectal excision (abTME).

**Methods:**

Patients who underwent taTME or abTME for stages I–III rectal cancer and who received an anastomosis were included. A retrospective analysis of a prospectively conducted database was performed. The primary endpoints were overall survival (OS), cancer-specific survival (CSS), and disease-free survival (DFS). Risk factors were adjusted by propensity score matching (PSM). The secondary endpoints were local recurrence rates and combined poor pathological outcomes.

**Results:**

From 2012 to 2020, a total of 189 patients underwent taTME, and 119 underwent abTME; patients were followed up for a mean of 54.7 (SD 24.2) and 78.4 (SD 34.8) months, respectively (*p* < 0.001). The 5-year survival rates after taTME and abTME were not significantly different after PSM: OS: 78.2% vs. 88.6% (*p* = 0.073), CSS: 87.4% vs. 92.1% (*p* = 0.359), and DFS: 69.3% vs. 80.9% (*p* = 0.104), respectively. No difference in the local recurrence rate was observed (taTME, *n* = 10 (5.3%); abTME, *n* = 10 (8.4%); *p* = 0.280). Combined poor pathological outcomes were more frequent after abTME (*n* = 36, 34.3%) than after taTME (*n* = 35, 19.6%) (*p* = 0.006); this difference was nonsignificant according to multivariate analysis (*p* = 0.404).

**Conclusion:**

taTME seems to be a good treatment option for patients with rectal cancer and is unlikely to significantly affect local recurrence or survival. However, further investigations concerning the latter are warranted.

**Trial registration:**

ClinicalTrials.gov (NCT0496910).

**Supplementary Information:**

The online version contains supplementary material available at 10.1007/s00384-023-04591-7.

## Purpose

In 2020, rectal cancer accounted for 3.8% of all new cancer diagnoses and 3.4% of all cancer-related deaths worldwide [1]. Surgical treatment of rectal cancer requires surgery along the anatomical and embryological planes [2]. This technique, called total mesorectal excision (TME), reduces the local recurrence rate and improves survival [3]. In the early 2000s, surgeons started to perform TME laparoscopically. The advantage of this approach was improved postoperative recovery and cosmesis. In experienced hands, similar oncological outcomes could be achieved [4, 5]. However, in patients with low rectal cancer, the laparoscopic approach and anastomosis are more difficult, resulting in a greater risk of positive circumferential margins (CRMs) [6] as well as a greater incidence of anastomotic leakage [7].

In 2009, Lacy et al. and Zorron et al. were the first to describe transanal TME (taTME) [8, 9]. This novel technique combines abdominal with transanal dissection under endoscopic vision. taTME enables better visualization of the mesorectal plane in the lower rectum and should result in a greater percentage of free CRM and improved specimen quality [10–12]. Furthermore, recent analyses of single- and multicenter data have shown a lower rate of anastomotic leakage [13, 14] as well as comparable short-term [10, 15] and long-term oncological outcomes [12, 13] after taTME compared to abdominal TME.

However, there is still controversy about the taTME in the current scientific literature. In Norway and the Netherlands, Wasmuth et al. and van Oostendorp et al. described a higher rate of anastomotic leakage and, even more disturbingly, a greater rate of local recurrence with multifocal growth patterns. Additionally, a high mortality rate and rather low disease-free survival rate have been reported [16–18]. In 2019, the Norwegian Colorectal Cancer Group imposed a moratorium on taTME because of the elevated local recurrence rate [19].

The aim of this study was to compare the long-term and short-term outcomes of taTME with those of anterior abdominal approaches (open, laparoscopic, and robotic) (abTME).

## Methods

### Patients

Patients who underwent elective TME followed by reconstruction with anastomosis for primary rectal cancer between January 2012 and December 2020 at the Cantonal Hospital of St. Gallen were enrolled in this study. The patients were identified retrospectively in the electronic hospital records and were divided into two cohorts: the intervention group, who underwent taTME, and the control group, who underwent abTME. The operating surgeon decided whether to perform taTME or abTME and which approach to use (e.g., open abTME after a previous complex abdominal operation).

The exclusion criteria were diagnoses other than rectal cancer, recurrent rectal cancer, partial mesorectal excision (PME), discontinuity resection (no anastomosis), incomplete TNM staging information, metastatic cancer, lack of follow-up, patients who declined to have their information included in the scientific data analysis, and patients under 18 years of age. All patients were asked to provide consent for scientific analysis of their data before inclusion in our clinic database. A “decline for scientific data analysis by patients” indicated that the patients either did not sign the consent or withdrew the approval later. Detailed information concerning patient selection can be found in the Supplementary Material, Figure S1.

The following patient characteristics were collected: age, sex, American Society of Anesthesiologists (ASA) classification, body mass index (BMI, kg/m^2^), (neo-) adjuvant therapy, and tumor height (assessed from the anal verge using a rigid rectoscope). Postoperative morbidity was classified according to the Clavien‒Dindo Classification [20]. Major postoperative morbidity was defined as a Clavien‒Dindo grade greater than 3a. All patients underwent regular surveillance follow-up, including an annual CT scan, according to the guidelines of the Swiss Society of Gastroenterology [21].

### Operative procedure, neoadjuvant and adjuvant treatment, and pathological assessment

All operations were performed by experienced colorectal surgeons. For both taTME and abTME, the inferior mesenteric artery was divided at its origin from the aorta, and the inferior mesenteric vein was divided at the inferior border of the pancreas. Dissection of the mesorectal plane was performed along the TME plane while considering the hypogastric nerve plexus, the gonadal vessels, and the ureters.

For abTME, complete dissection was performed from above, following embryological planes down to the pelvic floor, so all mesorectal fat was removed. In general, the lower rectum was divided using one or more linear staplers 1 cm above the sphincter according to the tumor height. A double stapling technique for anastomosis using a 28- to 31-mm circular stapler was performed. In case of the necessity of intersphincteric resection, the division was performed open followed by hand-sewn coloanal anastomosis. For abTME, one team performed the whole procedure. The first laparoscopic abTME was conducted on April 4, 2012, and the first robotic abTME was conducted on January 20, 2015. The study was designed to start at the beginning of 2012 to accommodate the learning curve for all minimally invasive procedures.

For taTME, a two-team synchronous approach involving the transanal and abdominal (always laparoscopic) approaches was performed, fashioning a tight closed purse-string suture from the endorectal clearly below the tumor, followed by complete rectotomy and a second purse string. Thorough transanal wash-out was performed before and after each purse string. After each wash-out, the gloves and swaps were changed to new ones. taTME was completed along the mesorectal plane under direct endoscopic vision in a rendezvous procedure with the abdominal team. taTME was introduced in 2013, and the first taTME was conducted on October 30, 2013.

For abTME and taTME, the anastomosis was checked for bleeding or leakage with a rectoscope. A protective ileostomy was placed through the musculus rectus abdominis at a preoperatively marked position.

Neoadjuvant and adjuvant therapy were administered as recommended by an interdisciplinary team meeting and included long-course radiochemotherapy, short-course radiotherapy, and/or chemotherapy.

The quality of the specimen after rectal cancer resection was assessed and graded as optimal, suboptimal, or poor according to the Quirke score as described by Phil Quirke [22, 23].

### Analyzed outcome measures and data collection

The primary outcomes were overall survival (OS; time from operation to death), cancer-specific survival (CSS; time from operation to death caused by rectal cancer; deaths due to other causes were censored), and disease-free survival (DFS; time from operation to local and/or systemic recurrence or death). The secondary outcomes were pathological quality parameters (resection margin (R), specimen quality, distance to circumferential resection margin (CRM), distance to distal resection margin (DRM), total number of lymph nodes harvested), postoperative morbidity (including anastomotic leakage as defined by the International Study Group of Rectal Cancer [24]), 30- and 90-day mortality, local recurrence (radiological and/or pathological evidence of recurrent disease in the small pelvis), and overall recurrence (radiological and/or pathological evidence of recurrent disease anywhere in the body). Furthermore, a combined poor pathological outcome was defined as one or more of the following: R1/2, specimen quality poorer than good, CRM smaller than 2 mm, or total number of lymph nodes harvested less than 12.

Patient data were collected from the electronic medical records. Data from rectal cancer patients were entered prospectively in a scientific, relational database. Missing data were sought after contacting the patients, their general practitioners, and gastroenterologists. Prospectively collected data were retrospectively analyzed.

### Statistical analysis

Statistical analyses were performed using R statistical software (www.r-project.org). A two-sided *p* value of less than 0.05 was considered statistically significant. Continuous data are expressed as the mean ± standard deviation (SD). Proportions were compared with the chi-square test, and continuous variables were compared with the *t* test and the Mann‒Whitney *U* test, as appropriate. For logistic, the Cox, and cumulative link regression analyses, *p* values were estimated by likelihood ratio tests, and 95% confidence intervals (CIs) were obtained by the Wald method [25, 26].

After initial univariate comparisons of the taTME and abTME groups in terms of baseline, staging, and treatment characteristics, logistic regression was applied to further evaluate the univariate and multivariate distributions of age (continuous), sex, body mass index (BMI, continuous), ASA group, UICC stage, cancer localization, neoadjuvant therapy, and year of operation (defined as the confounding variable set) in the two groups. Multivariate regression models were complemented by backward variable selection based on Akaike’s information criterion (AIC). Then, taTME and abTME were compared via univariate analyses in terms of short-term postoperative morbidity and mortality.

The quality of the pathological specimens evaluated using the Quirke score was statistically assessed by univariate descriptive analysis and by univariate and multivariate cumulative link ordinal regression analyses with and without adjustment for the same confounding variable set already used for logistic regression and accomplished by a backward variable selection procedure. The use of a cumulative link model for ordinal regression increases the statistical precision of the analysis of ordinal data such as the Quirke score and CRM [27]. The same analysis was repeated for the CRM as the dependent variable. The cumulative link ordinal regression analyses were performed with flexible thresholds using the R library ordinal. The threshold of 12 or more harvested regional lymph nodes was then assessed via univariate and multivariate logistic regression with and without adjustment for the confounding variable set and was accomplished via a backward variable selection procedure. The same analysis was ultimately applied to a combined short-term pathological outcome measure. An unfavorable outcome was defined as a Quirke score worse than good and/or a CRM smaller than 2 mm and/or R1 resection and/or less than 12 harvested lymph nodes. This combined outcome was assessed by univariate descriptive analysis and by univariate and multivariate logistic regression with and without adjustment for the confounding variable set and was accomplished by a backward variable selection procedure.

After descriptive analysis of the categorical local and overall recurrence rates, local and overall recurrence rates were analyzed as time-to-event data via the univariate Cox regression. In these analyses, deaths without previous recurrence were censored, and only recurrences were counted as events. The variance was estimated with the robust Huber sandwich estimator, and the proportional hazard assumption was checked by comparing the Schoenfeld residuals against time.

Overall, cancer-specific and disease-free survival were first analyzed via the unadjusted Cox regression with the robust Huber sandwich variance estimation, and the Schoenfeld residuals were compared against the time to control for a violation of the proportional hazard assumption. Given the relevant bias in the UICC stage and year of operation between the taTME and abTME groups, additional univariate and multivariate Cox regression analyses were performed with stratification for the UICC stage and year of operation with and without adjustment for the confounding variable set (without the stratification variables UICC stage and year of operation) and accomplished by a backward variable selection procedure.

To further address potential bias [28, 29], a full bipartite matching and weighting propensity score analysis was performed using the “Matching” R package [30] based on the confounding variable set and its interactions with the year of operation. The propensity score is described as a value between 0 and 1 ± standard deviation (SD). In this procedure, one-to-many patients from one group are matched in subclasses with one-to-many patients from the other group, and additional weights are assigned such that at the end of this procedure, there is a virtually similar propensity score in the two groups. Patients who underwent taTME (*n* = 43) but did not have a counterpart among the patients who underwent abTME and vice versa (*n* = 67) were excluded from this analysis. The baseline variables for the two groups were compared by multivariate logistic regression conditional on the subgroups identified by the propensity score matching and weighting (PSM) procedure. The variables adjusted for were age (continuous), sex, BMI (continuous), ASA group (≤ II, ≥ III), UICC stage, tumor location (< 6 cm, 6–12 cm, > 12 cm from the anal verge), neoadjuvant therapy, adjuvant therapy, and year of operation. The rationale for continuous instead of categorical variables was that the statistical precision is greater for a given degree of freedom.

Overall, cancer-specific and disease-free survival were assessed via the stratified Cox regression analyses by stratifying the subclasses according to the weights obtained via the PSM procedure with the robust Huber sandwich variance estimates for variance and controlling for a violation of the proportional hazard assumption by comparing the Schoenfeld residuals against time.

The complete analysis was repeated after excluding patients who underwent open or conversion surgery as a subgroup analysis.

### Ethics

The study was approved by the Ethics Committee of Eastern Switzerland (BASEC Nr. 2021–01259), registered at ClinicalTrials.gov (NCT0496910) and is compliant with the STROCSS guidelines.

## Results

### Baseline, staging, and treatment characteristics

A total of 457 patients underwent rectal resection for primary rectal cancer between 2012 and 2020. A total of 308 patients were enrolled in this study. A total of 189 (61.4%) of the enrolled patients underwent taTME, and 119 (38.6%) underwent abTME. The mean follow-up time was shorter in the taTME group than in the abTME group (54.7 (SD24.2) vs. 78.4 (SD 34.8) months, *p* < 0.001).

Patient characteristics are summarized in Table 1. The taTME group had significantly more male patients (*n* = 134 [70.9%] vs. *n* = 67 [56.3%], *p* = 0.009) and a greater mean BMI (27.0 vs. 24.8, *p* < 0.001) than the abTME group. taTME was performed or instructed mainly by the same surgeon responsible for the introduction of this new technique (*n* = 179 (94.7%)). Twenty-eight patients underwent open TME. Conversion was needed less often in the taTME group (*n* = 10 [5.3%] vs. *n* = 21 [17.6%]; *p* < 0.001). The UICC stage was distributed unevenly, with a clearly greater proportion of stage II patients among patients who underwent abTME (*p* = 0.029).

**Table 1:** Patient baseline, treatment, and staging characteristics (before matching)

**Variable**	**Label**	**abTME (*n* = 119)**	**taTME (*n* = 189)**	***p* value**^**d**^
Year of operation	To 2014	82 (68.9%)	18 (9.5%)	** < 0.001 C)**
	2015–2017	24 (20.2%)	89 (47.1%)	
	Since 2018	13 (10.9%)	82 (43.4%)	
Follow-up	Mean (SD)	78.4 (34.8)	54.7 (24.2)	** < 0.001 A)**
Age (years)	Mean (SD)	66.6 (11.8)	64.1 (10.9)	0.085 A)
Sex	Female	52 (43.7%)	55 (29.1%)	**0.009 C)**
	Male	67 (56.3%)	134 (70.9%)	
ASA classification	I/II	81 (68.1%)	141 (74.6%)	0.102 B)
	III	36 (30.3%)	48 (25.4%)	
	IV	2 (1.7%)	0 (0.0%)	
BMI (kg/m^2^)	< 30	108 (90.8%)	153 (81.0%)	**0.020 C)**
	≥ 30	11 (9.2%)	36 (19.0%)	
	Mean (SD)	24.8 (3.7)	27.0 (4.3)	** < 0.001 A)**
Tumor height	< 6 cm	23 (19.3%)	46 (24.3%)	0.092 C)
	6 to < 12 cm	82 (68.9%)	133 (70.4%)	
	12 to 16 cm	14 (11.8%)	10 (5.3%)	
Neoadjuvant therapy	No	43 (36.1%)	64 (33.9%)	0.275 B)
	Radiochemotherapy	55 (46.2%)	91 (48.1%)	
	Radiotherapy	16 (13.4%)	32 (16.9%)	
	Chemotherapy	5 (4.2%)	2 (1.1%)	
Adjuvant therapy	No	62 (52.1%)	112 (59.3%)	0.138 B)
	Radiochemotherapy	1 (0.8%)	6 (3.2%)	
	Chemotherapy	56 (47.1%)	71 (37.6%)	
Planned surgical access	Open	28 (23.5%)	0 (0.0%)	** < 0.001 B)**
	Laparoscopy	76 (63.9%)	189 (100.0%)	
	Robotic	15 (12.6%)	0 (0.0%)	
Conversion	No	98 (82.4%)	179 (94.7%)	** < 0.001 C)**
	Yes	21 (17.6%)	10 (5.3%)	
	Laparoscopy to open	16 (21.1%)^e^	10 (5.3%)^e^	
	Robotic to laparoscopy	4 (26.6%)^e^		
	Robotic to open	1 (6.7%)^e^		
pT category	T0	11 (9.2%)	22 (11.6%)	0.115 B)
	T1	6 (5.0%)	25 (13.2%)	
	T2	38 (31.9%)	60 (31.7%)	
	T3	58 (48.7%)	78 (41.3%)	
	T4a	3 (2.5%)	1 (0.5%)	
	T4b	3 (2.5%)	3 (1.6%)	
pN category	N0	83 (69.7%)	129 (68.3%)	0.766 C)
	N1	26 (21.8%)	47 (24.9%)	
	N2	10 (8.4%)	13 (6.9%)	
Grading	G1	1 (1.4%)^a^	9 (6.0%)^a^	**0.031**^**b**^** A)**
	G2	56 (77.8%)^a^	119 (79.3%)^a^	
	G3	15 (20.8%)^a^	22 (14.7%)^a^	
UICC stage	I	43 (36.1%)	90 (47.6%)	**0.029 C)**
	II	40 (33.6%)	39 (20.6%)	
	III	36 (30.3%)	60 (31.7%)	
TRG Dworak	Grad 0	3 (4.2%)^c^	10 (8.1%)^c^	**0.006**^**b**^** A)**
	Grad 1	31 (43.7%)^c^	46 (37.4%)^c^	
	Grad 2	26 (36.6%)^c^	40 (32.5%)^c^	
	Grad 3	3 (4.2%)^c^	8 (6.5%)^c^	
	Grad 4	8 (11.3%)^c^	19 (15.4%)^c^	
	No neoadjuvant radiotherapy	46 (38.7%)	65 (34.4%)	

*SD* standard deviation

A) Mann‒Whitney U test, B) chi-square test, MC simulated, C) chi-square test

^a^Missing values excluded

^b^only data in the test included: values not missing/therapy received

^c^Missing and not treated values excluded

^d^Significant values are bold

^e^Reference is surgical access in the corresponding TME group

The logistic regression models indicated that higher BMI and later year of operation were significant predictors of taTME according to univariate, multivariate, and stepwise analyses. A younger age was confirmed to be a significant predictor of taTME only in multivariate and stepwise analyses; male sex and lower UICC stage were confirmed to be significant predictors in univariate analyses; and neoadjuvant therapy was confirmed to be a significant predictor in multivariate and stepwise analyses (Table 2).

**Table 2:** Univariate, multivariate, and stepwise logistic regression analyses for taTME

**Variable**	**Label**	**Univariate OR (95% CI)**	*p* value*	**Multivariate OR (95% CI)**	*p* value*	**Stepwise selection OR (95% CI)**	*p* value*
Age	(Years)	0.98 (0.96–1.00)	0.059	0.94 (0.91–0.97)	** < 0.001**	0.94 (0.91–0.97)	** < 0.001**
Sex	F	Reference	**0.009**	Reference	0.143	Reference	0.134
	M	1.89 (1.17–3.06)		1.67 (0.84–3.34)		1.69 (0.85–3.36)	
BMI	(kg/m^2^)	1.15 (1.09–1.23)	** < 0.001**	1.16 (1.07–1.27)	** < 0.001**	1.16 (1.07–1.27)	** < 0.001**
ASA classification	I/II	Reference	0.215	Reference	0.791	-	-
	III/IV	0.73 (0.44–1.21)		1.11 (0.53–2.35)		-	-
UICC stage	I	Reference	**0.030**	Reference	0.090	Reference	0.093
	II	0.47 (0.26–0.82)		0.43 (0.18–0.99)		0.43 (0.19–0.99)	
	III	0.80 (0.46–1.38)		0.51 (0.23–1.11)		0.51 (0.23–1.12)	
Tumor height	< 6 cm	Reference	0.098	Reference	0.066	Reference	0.066
	6 to < 12 cm	0.81 (0.45–1.42)		0.80 (0.34–1.80)		0.80 (0.35–1.80)	
	12 to 16 cm	0.36 (0.13–0.92)		0.21 (0.05–0.81)		0.21 (0.05–0.82)	
Neoadjuvant therapy	No	Reference	0.684	Reference	**0.032**	Reference	**0.032**
	Yes	1.11 (0.68–1.78)		2.19 (1.07–4.61)		2.20 (1.07–4.61)	
Year of operation	To 2014	Reference	** < 0.001**	Reference	** < 0.001**	Reference	** < 0.001**
	2015–2017	16.89 (8.74–34.26)		37.64 (16.29–95.79)		37.15 (16.18–94.00)	
	Since 2018	28.74 (13.66–64.93)		68.39 (25.80–206.84)		67.90 (25.68–204.53)	

Univariate and multivariate logistic regressions were performed with additional stepwise variable selection from full multivariate logistic regression. A higher OR indicates greater odds for taTME than for abTME

Odds ratios (ORs) with 95% confidence intervals

*likelihood ratio test; significant values are shown in bold

### Operation time, short-term postoperative morbidity and mortality

The mean operation time was shorter in the taTME group than in the abTME group (247.1 min vs. 289.8 min, *p* < 0.001) (Table 3).

**Table 3:** Clinical, surgical, and pathological outcomes and survival rates

**Variable**	**Label**	**Total (*n* = 308)**	**abTME (*n* = 119)**	**taTME (*n* = 189)**	*p* value*
Operation time (minutes)	Mean (SD)	263.6 (76.7)	289.8 (78.1)	247.1 (71.1)	** < 0.001 A)**
Quirke score	Optimal	272 (88.3%)	99 (83.2%)	173 (91.5%)	0.076 B)
	Suboptimal	29 (9.4%)	16 (13.4%)	13 (6.9%)	
	Poor	7 (2.3%)	4 (3.4%)	3 (1.6%)	
	Optimal	272 (88.3%)	99 (83.2%)	173 (91.5%)	**0.031 A)**
	Suboptimal or poor	36 (11.7%)	20 (16.8%)	16 (8.5%)	
CRM	< 2 mm	19 (6.7)^a^	9 (8.6)^a^	10 (5.6)^a^	0.468^a^ B)
	≥ 2 mm	265 (93.3)^a^	96 (91.4)^a^	169 (94.4)^a^	
Resection margin	R0	300 (97.4%)	116 (97.5%)	184 (97.4%)	0.969 D)
	R1(or more)	8 (2.6%)	3 (2.5%)	5 (2.6%)	
	CRM positive	6 (75.0%)	3 (100%)	3 (60%)	
	DRM and CRM-positive	2 (25.0%)		2 (40%)	
Number of lymph nodes harvested	0 to 11	34 (11.0%)	20 (16.8%)	14 (7.4%)	**0.010 C)**
	≥ 12	274 (89.0%)	99 (83.2%)	175 (92.6%)	
	Mean (SD)	18.1 (6.5)	17.6 (6.7)	18.5 (6.3)	0.132 A)
Clavien‒Dindo grade	None	95 (30.8%)	30 (25.2%)	65 (34.4%)	0.656 C)
	Grade 1	33 (10.7%)	12 (10.1%)	21 (11.1%)	
	Grade 2	90 (29.2%)	39 (32.8%)	51 (27.0%)	
	Grade 3a	24 (7.8%)	11 (9.2%)	13 (6.9%)	
	Grade 3b	53 (17.2%)	23 (19.3%)	30 (15.9%)	
	Grade 4a	8 (2.6%)	3 (2.5%)	5 (2.6%)	
	Grade 4b	3 (1.0%)	1 (0.8%)	2 (1.1%)	
	Grade 5 (death)	2 (0.6%)	0 (0.0%)	2 (1.1%)	
Major complication	Clavien‒Dindo grade ≤ 3a	242 (78.6%)	92 (77.3%)	150 (79.4%)	0.669 B)
	Clavien‒Dindo grade > 3a	66 (21.4%)	27 (22.7%)	39 (20.6%)	
Laparotomy because of	No	296 (96.1%)	115 (96.6%)	181 (95.8%)	0.727 D)
Anastomotic leakage	Yes	12 (3.9%)	4 (3.4%)	8 (4.2%)	
30-day mortality	Yes	2 (0.6%)	0 (0.0%)	2 (1.1%)	0.376 D)
90-day mortality	Yes	5 (1.6%)	0 (0.0%)	5 (2.6%)	0.085 D)
Local recurrence (days)	Yes	20 (6.5%)	10 (8.4%)	10 (5.3%)	0.280 C)
	Mean (SD)	590.0 (356.5)	636.6 (415.7)	543.3 (301.0)	0.853 A)
Overall recurrence (days)	Yes	75 (24.4%)	36 (30.3%)	39 (20.6%)	0.056 C)
	Mean (SD)	615.7 (483.8)	628.6 (474.8)	603.8 (497.9)	0.771 A)
3-year survival rates	Overall (95% CI)		89.8% (84.6–95.5%)	93.0% (89.4–96.8%)	
	Cancer-specific (95% CI)		93.2% (88.7–97.9%)	96.2% (93.5–99.0%)	
	Disease-free (95% CI)		71.2% (63.5–79.9%)	77.7% (71.9–84.0%)	
5-year survival rates	Overall (95% CI)		77.2% (69.8–85.3%)	80.1% (73.8–87.0%)	
	Cancer-specific (95% CI)		84.6% (78.0–91.6%)	88.2% (82.9–93.8%)	
	Disease-free (95% CI)		62.3% (54.0–71.8%)	71.6% (65.0–78.9%)	

A) Mann‒Whitney *U* test, B) chi-square test, MC simulated, C) chi-square test, D) Mid-*p* test

*SD* standard deviation, *CI* confidence interval

^a^complete case analysis ignoring missing values in 14 and 10 patients who underwent taTME and abTME

*Significant values are in bold

The incidence of major postoperative morbidities after 30 days was similar in patients who underwent taTME or abTME (*n* = 39 (20.6%) vs. *n* = 27 (22.7%), *p* = 0.669). Anastomotic leakage requiring laparotomy was similar in both groups (*n* = 8 (4.2%) vs. *n* = 4 (3.4%), *p* = 0.727). There was no significant difference in 30-day mortality (*n* = 2 [1.1%] vs. *n* = 0 [0.0%]; *p* = 0.376) or 90-day mortality (*n* = 5 [2.6%] vs. *n* = 0 [0.0%]; *p* = 0.085) between the taTME and abTME groups (Table 3). Two patients died due to cardiac arrest, one patient died due to esophageal perforation (30 days), one patient died due to septic shock because of anastomotic leakage (30 days), and one cause of death remains unknown.

### Pathological outcome parameters

#### Microscopically noncomplete resections

Overall, there were *n* = 8 (2.6%) microscopically noncomplete resections (≥ R1), including *n* = 5 (2.6%) in patients who underwent taTME (3 CRM-positive and 2 CRM- and DRM-positive) and *n* = 3 (2.5%) after abTME (all CRM-positive) (*p* = 0.969) (Table 3).

#### Quirke score

Suboptimal or poor mesorectal quality was found less often after taTME than after abTME (*n* = 16 (8.5%) vs. *n* = 20 (16.8%), *p* = 0.031) (Table 3). According to the results of the cumulative link model ordinal regression, taTME (*p* = 0.029) and ASA I/II status (*p* = 0.047) were significantly associated with a better mesorectal quality according to univariate analysis, and lower BMI was significantly associated with better mesorectal quality according to univariate (*p* = 0.010) and stepwise analyses (*p* = 0.034).

#### Circular resection margin (CRM)

A CRM smaller than 2 mm was reported in *n* = 10 specimens (5.6% of all patients with reported data) after taTME and in *n* = 9 specimens (8.6% of all patients with reported data) after abTME (*p* = 0.468) (Table 3). According to the results of the ordinal regression analysis, a high UICC stage (*p* < 0.001 according to univariate, multivariate, and stepwise analyses) and a low tumor location (*p* = 0.028 according to multivariate analysis and *p* = 0.048 according to stepwise analysis) showed a significant association with a CRM smaller than 2 mm.

#### Number of harvested lymph nodes

The number of harvested lymph nodes was on average 18.1 in all patients. After taTME, a mean of 18.5 lymph nodes and after abTME, a mean of 17.6 lymph nodes were found (*p* = 0.132). A threshold of 12 or more lymph nodes was reached after taTME in 175 (92.6%) patients and after abTME in 99 (83.2%) patients (*p* = 0.010) (Table 3). According to our logistic regression, the effect of taTME on more than 12 lymph nodes retrieved was confirmed by univariate analysis (*p* = 0.012) but was not significant according to multivariate analysis (*p* = 0.213). A later year of operation was a significant factor according to univariate (*p* = 0.003) and stepwise analyses (*p* = 0.003) but not according to multivariate analysis (*p* = 0.130).

#### Combined histopathological outcomes

Of the 284 patients for whom all data were available, 71 (25.0%) had unfavorable combined short-term pathological outcomes (Quirke score worse than good and/or a CRM smaller than 2 mm and/or R1 and/or less than 12 lymph nodes), including 35 (19.6%) patients after taTME and 36 (34.3%) patients after abTME. Univariate analysis revealed worse combined poor short-term pathological outcomes in patients who underwent abTME than in those who underwent taTME (*p* = 0.006); the difference was nonsignificant according to multivariate analysis (*p* = 0.404). The only other significant factor associated with a good combined short-term pathological outcome was a later year of operation according to univariate (*p* = 0.004) and stepwise analyses (*p* = 0.004) (Table 4).

**Table 4:** Univariate, multivariate, and stepwise logistic regression analyses for unfavorable histopathological outcomes (*n* = 284)

**Variable**	**Lab**	**Univariate**		**Multivariate**		**Stepwise**	
		**OR (95% CI)**	*p* value*	**OR (95% CI)**	*p* value*	**OR (95% CI)**	*p* value*
Treatment factor	abTME	Reference	**0.006**	Reference	0.404	-	-
	taTME	0.47 (0.27–0.80)		0.72 (0.34–1.56)		-	-
Age	< 65	Reference	0.945	Reference	0.770	-	-
	≥ 65	1.02 (0.59–1.76)		1.09 (0.61–1.98)		-	-
Sex	F	Reference	1.000	Reference	0.511	-	-
	M	1.00 (0.57–1.78)		1.23 (0.67–2.30)		-	-
BMI		0.97 (0.90–1.03)	0.301	0.99 (0.92–1.06)	0.799	-	-
ASA classification	I/II	Reference	0.155	Reference	0.175	Reference	0.156
	III/IV	1.52 (0.85–2.69)		1.53 (0.82–2.82)		1.54 (0.85–2.72)	
UICC stage	I	Reference	0.741	Reference	0.973	-	-
	II	1.26 (0.65–2.43)		1.04 (0.51–2.10)		-	-
	III	0.98 (0.52–1.86)		0.95 (0.48–1.87)		-	-
Tumor height	< 6 cm	Reference	0.371	Reference	0.240	-	-
	6 to < 12 cm	0.63 (0.34–1.21)		0.57 (0.29–1.13)		-	-
	12 to 16 cm	0.62 (0.18–1.84)		0.50 (0.14–1.58)		-	-
Neoadjuvant therapy	No	Reference	0.381	Reference	0.662	-	-
	Yes	1.29 (0.73–2.35)		1.15 (0.62–2.19)		-	-
Year of operation	To 2014	Reference	**0.004**	Reference	0.130	Reference	**0.004**
	2015–2017	0.45 (0.24–0.85)		0.54 (0.24–1.19)		0.46 (0.24–0.88)	
	Since 2018	0.33 (0.16–0.66)		0.41 (0.16–0.98)		0.33 (0.16–0.65)	

Complete case analysis omits 24 of 308 patients with missing data for the circular resection margin

Odds ratios (ORs) with 95% confidence intervals

Univariate and multivariate logistic regression analyses with additional stepwise variable selection from the multivariate model for unfavorable histopathological outcomes. Unfavorable histopathological outcome was defined as a Quirke score worse than good and/or a CRM smaller than 2 mm and/or R1 resection and/or less than 12 harvested lymph nodes. A higher odds ratio indicates greater odds of an unfavorable histopathological outcome

*likelihood ratio test; significant values are shown in bold

### Local and overall recurrence

**Fig. 1:**
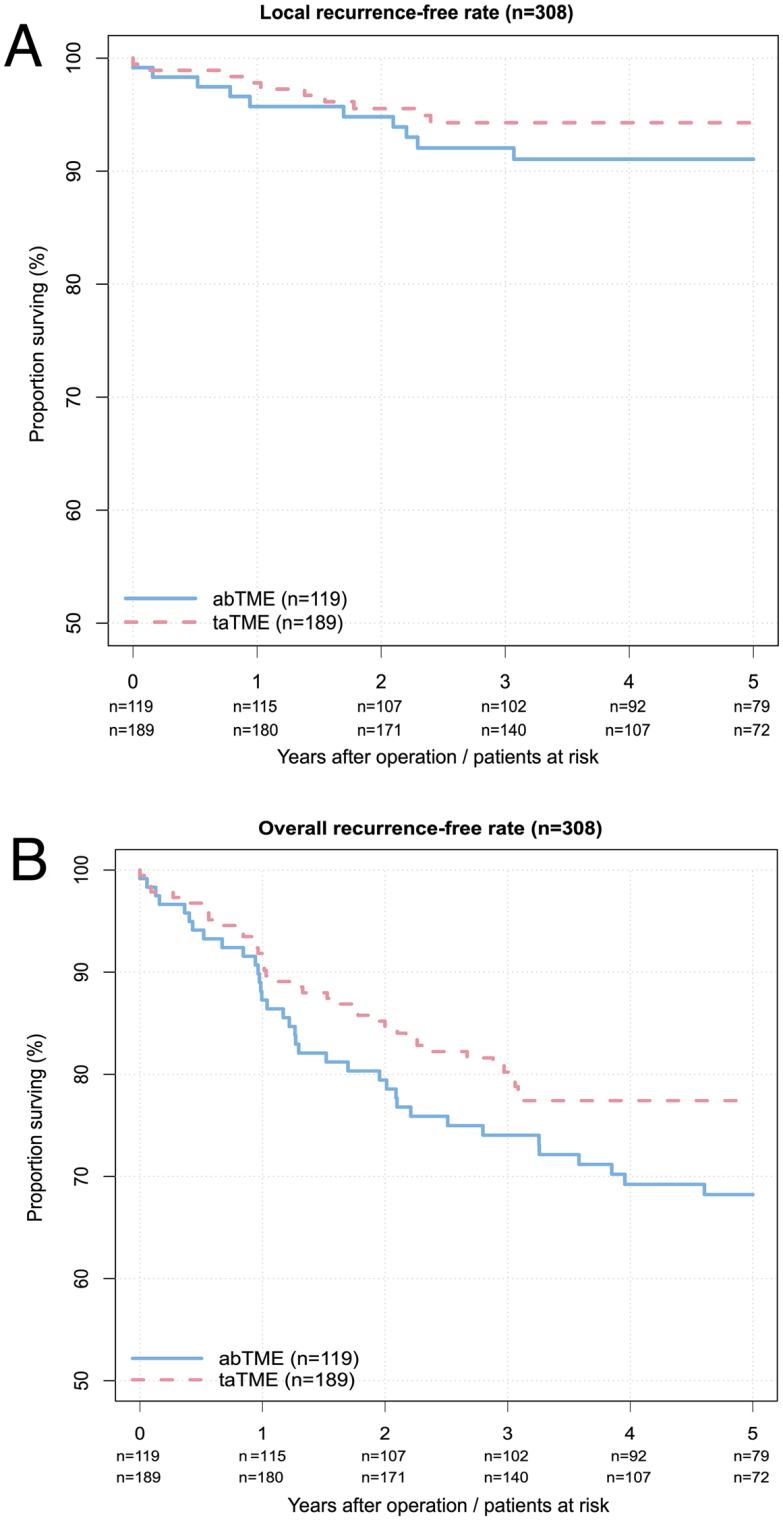
The univariate Cox regression analysis of patient outcomes after treatment with taTME (red dashed line) or abTME (blue line) (*n* = 308) unadjusted for multivariate or PSM analysis. **A** Local recurrence-free rate (*p* = 0.341*); **B** overall recurrence-free rate (*p* = 0.130*) *calculated by likelihood ratio tests

There were no significant differences in local recurrence rates (taTME, *n* = 10 (5.3%) vs. abTME, *n* = 10 (8.4%), *p* = 0.280) or overall recurrence rates (*n* = 39 (20.6%) vs. *n* = 36 (30.3%), *p* = 0.056) (Table 3). taTME did not influence the local recurrence-free rate (HR = 0.65; 95% CI: 0.27–1.57; *p* = 0.341) (Fig. 1A) or the overall recurrence-free rate (HR = 0.70; 95% CI: 0.45–1.11; *p* = 0.130) (Fig. 1B) (only recurrences were counted; deaths without recurrence were censored) over time. No local recurrences with multifocal growth patterns were observed in either group.

### Overall survival, cancer-specific survival, and disease-free survival, univariate and multivariate analyses

#### taTME; abTME: univariate analysis

Univariate analysis revealed the following 5-year overall, cancer-specific and disease-free survival rates after treatment with taTME and abTME:

- OS: 80.1% (95% CI: 73.8–87.0%) vs. 77.2% (95% CI: 69.8–85.3%)
- CSS: 88.2% (95% CI: 82.9–93.8%) vs. 84.6% (95% CI: 78.0–91.6%)
- DFS:71.6% (95% CI: 65.0–78.9%) vs. 62.3% (95% CI: 54.0–71.8%)

**Fig. 2:**
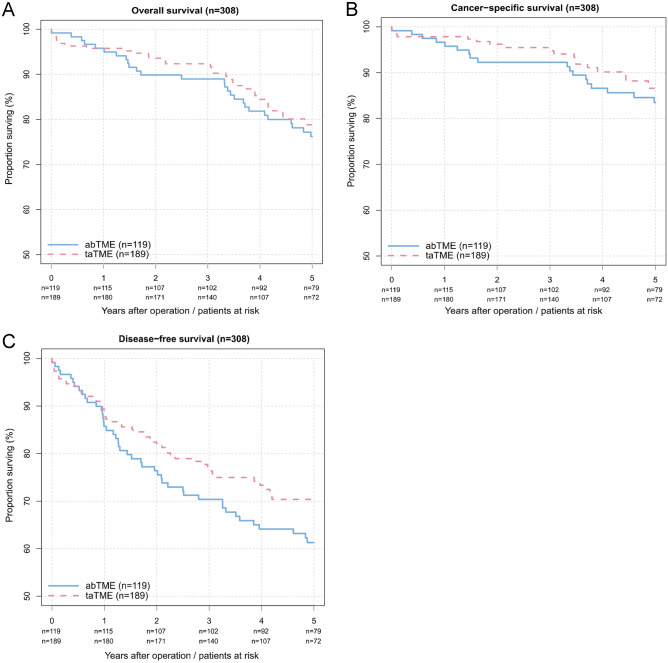
The univariate Kaplan‒Meier survival curves of patients after taTME (red dashed line) or abTME (blue line) (*n* = 308) unadjusted for multivariate or PSM analysis. **A** Overall survival (*p* = 0.723*); **B** cancer-specific survival (*p* = 0.560*); **C** disease-free survival (*p* = 0.204*) *calculated by likelihood ratio tests

The univariate and unadjusted survival curves are shown in Fig. 2.

In the univariate analysis, overall survival (HR = 0.92 (95% CI: 0.57 − 1.47), *p* = 0.723), cancer-specific survival (HR = 0.83 (95% CI: 0.45 − 1.54), *p* = 0.560), and disease-free survival (HR = 0.77 (95% CI: 0.52 − 1.15), *p* = 0.204) rates showed no significant difference after taTME or abTME. There was no evidence supporting the violation of the proportional hazard assumption (*p* = 0.360, *p* = 0.160, and *p* = 0.991).

#### taTME; abTME: the stratified Cox regression analyses (univariate, stepwise, and multivariate)

In the univariate and multivariate Cox regression analyses of the whole patient cohort (*n* = 308) stratified by UICC stage and year of operation with additional variable selection (stepwise analysis) (Table 5), taTME was associated with similar overall survival according to the univariate analysis (HR = 1.78 (95% CI: 0.79–4.03), *p* = 0.125) and with worse overall survival according to the multivariate analysis (HR = 2.81 (95% CI: 1.18–6.72), *p* = 0.012) and after variable selection (HR = 2.26 (95% CI: 0.94–5.45), *p* = 0.043). taTME was associated with similar cancer-specific survival rates according to univariate analysis (HR = 1.97 (95% CI: 0.59–6.57), *p* = 0.207) and after variable selection (HR = 2.32 (95% CI: 0.67–8.10), *p* = 0.136) and worse survival in the multivariate analysis (HR = 3.22 (95% CI: 0.95–10.94), *p* = 0.043). taTME was associated with similar disease-free survival rates according to univariate (HR = 1.23 (95% CI: 0.68–2.20), *p* = 0.498) and multivariate analyses (HR = 1.71 (95% CI: 0.92–3.21), *p* = 0.099) and was not an influential factor in the variable selection procedure.

**Table 5:** The univariate, multivariate, and stepwise Cox regression analyses stratified by year of operation and UICC stage for overall, cancer-specific, and disease-free survival

**Variable**	**Label**	**Univariate**		**Multivariate**		**Stepwise**	
		**HR (95% CI)**	*p* value*	**HR (95% CI)**	*p* value*	**HR (95% CI)**	*p* value*
**Overall survival**							
Treatment factor	abTME	Reference	0.125	Reference	**0.012**	Reference	**0.043**
	taTME	1.78 (0.79–4.03)		2.81 (1.18–6.72)		2.26 (0.94–5.45)	
Age		1.05 (1.02–1.07)	** < 0.001**	1.04 (1.01–1.06)	**0.008**	-	-
Sex	F	Reference	0.487	Reference	0.353	-	-
	M	0.84 (0.51–1.36)		0.77 (0.47–1.24)		-	
BMI		0.98 (0.92–1.04)	0.459	0.96 (0.91–1.02)	0.274	-	-
ASA classification	I/II	Reference	** < 0.001**	Reference	**0.009**	Reference	** < 0.001**
	III/IV	2.78 (1.74–4.44)		2.24 (1.28–3.91)		2.78 (1.64–4.70)	
Tumor height	< 6 cm	Reference	0.121	Reference	0.161	Reference	0.064
	6 to < 12 cm	0.56 (0.34–0.92)		0.57 (0.34–0.96)		0.50 (0.31–0.83)	
	12 to 16 cm	0.61 (0.28–1.34)		0.89 (0.37–2.13)		0.74 (0.31–1.78)	
Neoadjuvant therapy	No	Reference	0.946	Reference	0.553	-	-
	Yes	1.02 (0.62–1.68)		1.19 (0.73–1.95)		-	
Adjuvant therapy	No	Reference	**0.002**	Reference	0.106	Reference	**0.021**
	Yes	0.41 (0.25–0.69)		0.60 (0.35–1.03)		0.50 (0.29–0.87)	
**Cancer-specific survival**							
Treatment factor	abTME	Reference	0.207	Reference	**0.043**	Reference	0.136
	taTME	1.97 (0.59–6.57)		3.22 (0.95–10.94)		2.32 (0.67–8.10)	
Age		1.02 (1.00–1.05)	0.109	1.02 (0.99–1.05)	0.273	-	-
Sex	F	Reference	0.173	Reference	0.192	-	-
	M	0.63 (0.33–1.19)		0.60 (0.30–1.20)		-	
BMI		0.92 (0.84–1.01)	0.076	0.90 (0.83–0.99)	**0.032**	-	-
ASA classification	I/II	Reference	**0.012**	Reference	**0.010**	Reference	**0.003**
	III/IV	2.38 (1.29–4.39)		2.89 (1.40–5.99)		2.89 (1.52–5.51)	
Tumor height	< 6 cm	Reference	0.100	Reference	0.078	Reference	**0.042**
	6 to < 12 cm	0.46 (0.24–0.90)		0.50 (0.24–1.03)		0.41 (0.21–0.81)	
	12 to 16 cm	0.97 (0.40–2.36)		1.59 (0.53–4.83)		1.10 (0.41–2.96)	
Neoadjuvant therapy	No	Reference	0.463	Reference	0.283	-	-
	Yes	1.33 (0.63–2.84)		1.56 (0.76–3.19)		-	
Adjuvant therapy	No	Reference	0.328	Reference	0.985	-	-
	Yes	0.68 (0.33–1.41)		0.99 (0.50–1.97)		-	
**Disease-free survival**							
Treatment factor	abTME	Reference	0.498	Reference	0.099	-	-
	taTME	1.23 (0.68–2.20)		1.71 (0.92–3.21)		-	
Age		1.03 (1.01–1.05)	**0.004**	1.02 (1.00–1.04)	0.117	-	-
Sex	F	Reference	0.113	Reference	0.078	Reference	0.070
	M	0.71 (0.47–1.06)		0.66 (0.43–1.01)		0.67 (0.44–1.02)	
BMI		0.98 (0.94–1.03)	0.540	0.98 (0.93–1.02)	0.363	-	-
ASA classification	I/II	Reference	**0.001**	Reference	**0.029**	Reference	** < 0.001**
	III/IV	2.06 (1.40–3.04)		1.77 (1.11–2.81)		2.14 (1.44–3.18)	
Tumor height	< 6 cm	Reference	0.244	Reference	0.158	-	-
	6 to < 12 cm	0.67 (0.42–1.06)		0.68 (0.43–1.09)		-	
	12 to 16 cm	0.91 (0.47–1.73)		1.23 (0.58–2.60)		-	
Neoadjuvant therapy	No	Reference	0.719	Reference	0.836	-	-
	Yes	0.92 (0.59–1.44)		0.95 (0.61–1.48)		-	
Adjuvant therapy	No	Reference	**0.009**	Reference	0.166	-	-
	Yes	0.53 (0.35–0.82)		0.69 (0.43–1.10)		-	

A higher hazard ratio expresses a higher risk for unfavorable survival

*HR* hazard ratio, *CI* confidence interval

*Significant values are in bold

#### Other risk factors found in the stratified Cox regression analyses

Univariate, multivariate, and stepwise Cox regression analyses stratified by UICC stage and year of operation indicated that BMI, age, ASA classification, tumor height, and adjuvant therapy had significant associations with OS, CSS, and/or DFS (Table 5).

### Subgroup analysis after exclusion of open or converted operations

The results obtained while analyzing the complete study group were confirmed in a subgroup analysis repeating the complete analysis of 253 patients after the exclusion of 55 patients who underwent open or converted operations. The results of the logistic regression analyses for unfavorable histopathological outcomes and the results of the Cox regression analyses for survival in this subgroup analysis are shown in the Supplementary Material, Table S1 and Table S2.

### Overall survival, cancer specific survival, and disease-free survival after propensity score matching

#### Calculation of propensity scores; matched population

The risk factors of age, sex, BMI, ASA group, UICC stage, tumor location, neoadjuvant therapy, adjuvant therapy, and year of operation were evenly distributed after propensity score matching in the two groups, and none of them showed a significantly different distribution in univariate or multivariate conditional logistic regression after PSM. A well-matched sample of both groups (taTME, *n* = 146; abTME, *n* = 52) was generated by propensity score matching (propensity score before matching: 0.300 ± 0.281 abTME vs. 0.841 ± 0.168 taTME (*p* < 0.001); propensity score after matching: 0.796 ± 0.168 abTME vs. 0.795 ± 0.165 taTME (*p* = 0.982)). The patient characteristics were well adjusted after PSM (Table 6), and to attain that, 43 patients in the taTME group and 67 patients in the abTME group had to be removed from the analysis. This approach resulted in a rather small sample of 52 patients in the abTME cohort.

**Table 6:** Patient baseline characteristics after PSM (*n* = 198)

**Variable**	**Label**	**abTME**	**taTME**	***p* value**
Age (years)	Age	65.6 ± 7.2	66.9 ± 9.8	0.985
Sex	F	8.5 (16.4%)	46 (31.5%)	0.734
	M	43.5 (83.6%)	100 (68.5%)	
BMI (kg/m^2)	BMI	25.2 ± 3.3	26.2 ± 3.9	0.829
ASA classification	I/II	37.9 (72.9%)	106 (72.6%)	0.881
	III/IV	14.1 (27.1%)	40 (27.4%)	
UICC-stage	I	25.8 (49.7%)	65 (44.5%)	0.989
	II	7.2 (13.9%)	31 (21.2%)	
	III	18.9 (36.4%)	50 (34.2%)	
Tumor height	< 6 cm	9.1 (17.4%)	34 (23.3%)	0.984
	6 to < 12 cm	41.6 (80.1%)	103 (70.5%)	
	12 to 16 cm	1.3 (2.5%)	9 (6.2%)	
Neoadjuvant	No	20.3 (39.0%)	54 (37.0%)	0.976
therapy	Yes	31.7 (61.0%)	92 (63.0%)	
Adjuvant therapy	No	25.8 (49.6%)	84 (57.5%)	0.949
	Yes	26.2 (50.4%)	62 (42.5%)	
Year of operation	Years	2016.5 ± 1.7	2016.7 ± 1.8	0.852

Multivariate logistic regression conditional on the subgroups obtained by propensity score matching (PSM) and weighting

*n* (%), mean ± standard deviation

#### taTME; abTME: propensity score-adjusted analysis

**Fig. 3:**
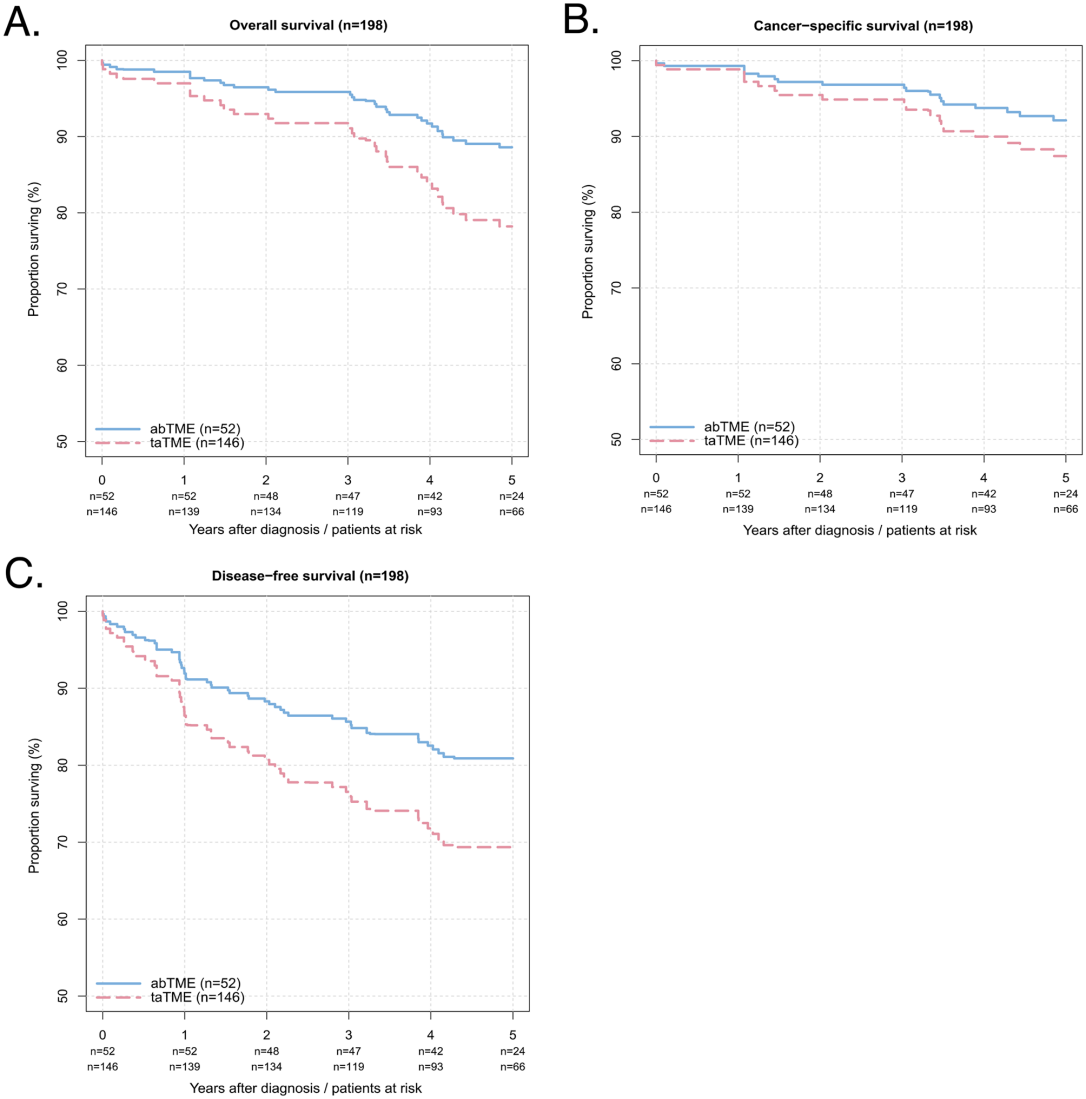
The propensity score-adjusted Kaplan‒Meier survival curves of patients after taTME (red dashed line) or abTME (blue line) (*n* = 198). **A** Overall survival (*p* = 0.073*); **B** cancer-specific survival (*p* = 0.359*); **C** disease-free survival (*p* = 0.104*) *calculated by likelihood ratio tests

Using the weights and subgroups obtained by PSM, no significant influence of taTME on the 5-year survival rates of OS (HR = 2.03 (95% CI: 0.78 − 5.26), *p* = 0.073), CSS (HR = 1.64 (95% CI: 0.40 − 6.75), *p* = 0.359), or DFS (HR = 1.73 (95% CI: 0.82 − 3.63), *p* = 0.104) was found after PSM, as shown in Fig. 3:

- OS: 78.2% (95% CI: 71.1–86.0%) vs. 88.6% (95% CI: 78.6–99.8%)
- CSS:87.4% (95% CI: 81.2–94.1%) vs. 92.1% (95% CI: 82.2–100.0%)
- DFS:69.3% (95% CI: 61.6–78.1%) vs. 80.9% (95% CI: 68.4–95.7%)

## Discussion

The value and safety of taTME in patients requiring rectum resection for the treatment of rectal cancer remain unclear. In this study, despite accounting for the learning curve for taTME, there were no significant differences between the taTME and abTME cohorts in terms of OS, CSS, or DFS in the 5-year unadjusted and propensity score-adjusted survival analyses. Furthermore, local recurrence was not significantly more common after taTME than after abTME. The pathological specimen analysis revealed better quality after taTME in univariate analysis but not in multivariate analysis.

The influence of a new therapy on survival is the most important criterion in cancer treatment. However, data on long-term survival after taTME are still sparse [12]. A previous Dutch publication reported a higher rate of local recurrence after taTME, thus raising concerns that this might translate into worse survival [17]. The survival data shown in this study do not support these concerns. The 5-year survival rates observed herein for both the taTME and abTME groups were similar to the survival rates reported in the ACOSOG Z6051 and ALaCaRT randomized trials [31, 32]. Furthermore, 5-year OS of 80.1% after taTME and 77.2% after abTME and the 5-year CSS rates of 88.2.3% and 84.6% compare well with the results reported in large databases such as SEER [33].

Only a few retrospective trials have compared long-term survival between patients who underwent taTME and patients who underwent abTME [34]. The majority of these trials showed no significant difference in survival between patients treated with taTME or abTME, consistent with the results found here [35, 36].

An early observed elevated rate of local recurrence compared with patients from the national registry was the main reason why the Norwegian Colorectal Cancer Group imposed a moratorium on taTME [19]. Additionally, a high rate of local recurrence (10%) with a high frequency of multifocal growth patterns (66.7%) was detected in the first 120 patients during an implementation program for taTME in the Netherlands [17]. Local recurrences were not significantly more common and did not occur earlier after taTME than after abTME in this study. Moreover, no multifocal growth pattern was observed. The significantly shorter mean follow-up of 54.7 vs. 78.4 months in the taTME group could explain the lack of increase in local recurrence rates. This seems unlikely, as no difference in the calculated local recurrence rate was observed over time (Fig. 1). The clearly greater number of UICC stage II tumors in the abTME group is a reasonable explanation for the approximately 10% greater rate of overall recurrence reported in this study. Unfortunately, the number of events was not high enough (at least 10 events are needed for one degree of freedom) to perform multivariate analysis. Therefore, this assumption could not be verified.

Before Wasmuth and van Oostendrop published their disturbing results, many studies reported that recurrence rates did not increase after taTME [12–14]. After the two worrisome publications, further studies looked even harder at the issue. Fortunately, elevated local recurrence rates were not observed in recent studies, such as the case series published by Simo and Volkel [10, 37], the retrospective multicenter study published by Roodbeen [38], the comparative studies published by Munini and Zeng [34, 35], the surveys published by the national database of Denmark and the international taTME registry [39, 40], and a new meta-analysis [36].

The reasons for the nonelevated local recurrence rate might be the structured implementation and the operative technique used to avoid cancer cell spillage. Keeping structured introduction and the prevention of dissemination of cancer cells in mind, the equal survival and local oncological outcomes seem to justify the performance of taTME. However, this is only valid until further randomized prospective controlled trials, such as the GRECCAR 11 and COLOR III studies, assess the short- and long-term outcomes of taTME [41, 42].

The new taTME procedure was relatively safe and did not increase short-term morbidity. Mortality was elevated after 90 days, but statistical analysis did not indicate a significant increase. Although the increase in mortality after taTME is likely coincidental, this is still worrisome, and this issue must be monitored closely in the future.

In this study, morbidity was comparable between the groups, although taTME was performed more often in patients with mid- and low rectal cancer who had a higher risk of intra- and postoperative complications [43]. Previous studies have reported the advantages of taTME regarding intra- and postoperative complications, including a decreased rate of anastomotic leakage [10, 44]. However, other studies have reported complication rates for patients receiving taTME that exceed those previously observed for patients receiving abTME [45, 46]. There have been reports of significantly greater leakage rates after taTME than after abTME [16, 47]. However, most relevant studies and recent meta-analyses have shown similar morbidity values after the two procedures [34, 36, 48, 49].

The specimen quality, in terms of the Quirke score, free CRM, and number of lymph nodes, is an important indicator of oncological outcome after TME [50]. The pathological specimen quality and completeness of resection seem to be slightly superior after taTME. Combined poor pathological outcome and the Quirke score, as well as the number of patients with more than 12 lymph nodes harvested, were significantly better in univariate analysis after taTME than after abTME. A reason for the greater number of lymph nodes found might be the ex vivo intra-arterial injection of indigo carmine, which is performed routinely in taTME patients. This finding was described by Widmann et al*.* in a previous article involving a different patient cohort from our hospital [51]. Furthermore, better visualization of the mesorectal plane in the taTME might lead to better specimen quality [52], as described in additional studies [13, 53, 54]. However, the better pathological outcome did not result in better survival or less local recurrence. The effect of higher specimen quality was nonsignificant in the multivariate analysis herein. Accordingly, the quality of the specimen was not significantly better after taTME in a recent meta-analysis and in a large cohort study [36, 49]. The parameter influencing combined poor pathological outcomes, which remained significant through logistic regression analysis, was the year of operation. A clear improvement was shown if the patient underwent surgery later, likely reflecting the learning curve of surgeons. taTME is a highly complex procedure that should be introduced at an institution and learned by surgeons via a structured pathway [55]. Even for surgeons well trained in laparoscopic TME, the learning curve for taTME seems to be at least 40 procedures before some proficiency develops. If this fact is neglected and structured training is not performed properly, worse outcomes may be observed [56].

### Strengths and limitations

A strength of this study is its thorough and long follow-up period, especially for the abTME group. The quality of the presented data was excellent (> 90% complete data), and no loss to follow-up occurred. Furthermore, a learning curve was included for taTME and laparoscopic and robotic abTME.

The main limitations of this study were its retrospective design, the absence of randomization, the clearly shorter follow-up in the taTME group, and the lack of differentiation among open, laparoscopic, and robotic TME patients in the abTME group. The selection of the procedure was performed by the operating surgeon according to the patient to be operated on and the preferences of the surgeon. This might have introduced some selection bias. However, most of the introduced differences in baseline parameters were corrected by propensity score matching, ensuring a balanced analysis.

Another important shortcoming is the limited statistical power, especially for dichotomous outcomes, which was low to moderate at best [57, 58]. This was caused by the necessity to exclude a relatively high number of patients for good propensity score matching, which resulted in a rather small sample, especially in the abTME group, regardless of the initial large study cohort of 308 patients.

The aim of this study was to compare traditional anterior access for TME with the newly developed posterior approach used for taTME, regardless of the invasiveness of the abdominal approach. The inclusion of patients with an open TME could have introduced bias in an otherwise minimally invasive cohort. On the other hand, the subgroup analysis performed after the exclusion of these patients showed no difference in the results and reduced the power of the analysis. Therefore, patients who underwent surgery via open access were included in this study. Moreover, other studies assessing the long-term oncological outcomes of open and laparoscopic surgery for rectal cancer have shown no differences related to the method of access [7, 59, 60].

## Conclusion

taTME seems to be safe and to have comparable long-term survival to abTME. However, although not significant, there were reductions in long-term survival (OS and DFS) of up to 10% in patients receiving taTME. This finding requires further investigation, particularly given that the statistical power to detect such differences as significant was only moderate. Therefore, taTME should be performed only at specialized centers for treating rectal cancer. A structured implementation seems to be essential. If the technique is introduced accordingly, this operation results in excellent specimen quality even in a population with a high proportion of obese men and low tumor locations.

Whether taTME becomes the standard of care for rectal cancer is not yet clear.

## Supplementary Information

Below is the link to the electronic supplementary material.

Supplementary file1 (TIFF 355605 KB)

Supplementary file2 (DOCX 47 KB)

Supplementary file3 (DOCX 27 KB)

## Data Availability

The anonymized patient data that support the findings of this study are available and can be obtained from the corresponding author, LM, upon reasonable request.
