# Supplementary material for: Is survival after transanal total mesorectal excision (taTME) worse than that after traditional total mesorectal excision? A retrospective propensity score-adjusted cohort study
Source: Int J Colorectal Dis. 2024 Feb 20;39(1):28. doi: 10.1007/s00384-023-04591-7 (PMC10879364; doi:10.1007/s00384-023-04591-7)
Supplement: Supplementary file 2 — Supplementary file2 (DOCX 47 KB) [file 384_2023_4591_MOESM2_ESM.docx]

**Table S1 Univariate, multivariate****, and stepwise logistic regression analysis for unfavorable** **histopathologic outcome in the subgroup analysis after exclusion of 55 patients with open or conversion surgery**

| **Variable** | **Lab** | **univariate** |  | **multivariate** |  | **stepwise** |  |
| --- | --- | --- | --- | --- | --- | --- | --- |
|  |  | **OR (95% CI)** | ***p* value***** | **OR (95% CI)** | ***p* value***** | **OR (95% CI)** | ***p* value***** |
| Treatment Factor | abTME | Reference | **0.032** | Reference | 0.488 | - | - |
|  | taTME | 0.49 (0.26-0.94) |  | 0.73 (0.30-1.81) |  | - | - |
| Age | < 65 | Reference | 0.799 | Reference | 0.556 | - | - |
|  | ≥ 65 | 1.08 (0.59-2.01) |  | 1.22 (0.63-2.39) |  | - | - |
| Sex | F | Reference | 0.276 | Reference | 0.134 | Reference | 0.137 |
|  | M | 1.45 (0.75-2.95) |  | 1.74 (0.85-3.77) |  | 1.72 (0.85-3.66) |  |
| BMI |  | 1.00 (0.93-1.07) | 0.913 | 1.01 (0.94-1.10) | 0.717 | - | - |
| ASA classification | I/II | Reference | 0.195 | Reference | 0.196 | Reference | 0.156 |
|  | III/IV | 1.57 (0.79-3.04) |  | 1.62 (0.78-3.31) |  | 1.68 (0.82-3.40) |  |
| UICC stage | I | Reference | 0.645 | Reference | 0.886 | - | - |
|  | II | 1.11 (0.51-2.34) |  | 1.09 (0.48-2.43) |  | - | - |
|  | III | 0.76 (0.37-1.56) |  | 0.88 (0.40-1.89) |  | - | - |
| Tumor height | < 6 cm | Reference | 0.349 | Reference | 0.111 | Reference | 0.128 |
|  | 6 to < 12 cm | 0.59 (0.29-1.22) |  | 0.43 (0.20-0.95) |  | 0.45 (0.21-0.97) |  |
|  | 12 to 16 cm | 0.76 (0.19-2.59) |  | 0.53 (0.12-2.01) |  | 0.58 (0.14-2.12) |  |
| Neoadjuvant therapy | No | Reference | 0.841 | Reference | 0.965 | - | - |
|  | Yes | 1.07 (0.57-2.06) |  | 1.02 (0.51-2.09) |  | - | - |
| Year operated | to 2014 | Reference | **0.009** | Reference | 0.059 | Reference | **0.002** |
|  | 2015–2017 | 0.44 (0.21-0.91) |  | 0.42 (0.16-1.07) |  | 0.37 (0.17-0.79) |  |
|  | since 2018 | 0.29 (0.13-0.64) |  | 0.28 (0.09-0.80) |  | 0.23 (0.10-0.54) |  |

Complete case analysis omnibus data for 18 of 253 patients with missing data for the circular resection margin
Odds ratios (OR) with 95% confidence interval
Univariate and multivariate logistic regression analysis with additional stepwise variable selection from multivariable model on unfavorable histopathologic outcomes. An unfavorable histopathologic outcome was defined as a Quirke Score worse than good and/or a CRM smaller than 2 mm and/or R1 resection and/or less than 12 harvested lymph nodes. A higher odds ratio indicates greater odds of an unfavorable histopathologic outcome
* - likelihood ratio tests, significant values are bold
