# Supplementary material for: Is survival after transanal total mesorectal excision (taTME) worse than that after traditional total mesorectal excision? A retrospective propensity score-adjusted cohort study
Source: Int J Colorectal Dis. 2024 Feb 20;39(1):28. doi: 10.1007/s00384-023-04591-7 (PMC10879364; doi:10.1007/s00384-023-04591-7)
Supplement: Supplementary file 3 — Supplementary file3 (DOCX 27 KB) [file 384_2023_4591_MOESM3_ESM.docx]

**Table S2 Univariate, multivariate, and stepwise Cox regression analysis stratified for year of operation and UICC stage of overall, cancer-specific, and disease-free survival in the subgroup analysis after excluding 55 patients who underwent open or conversion surgery**

| **Variable** | **Label** | **univariate** | |  | | **multivariate** | |  | | **stepwise** | |  | |  |
| --- | --- | --- | --- | --- | --- | --- | --- | --- | --- | --- | --- | --- | --- | --- |
|  |  | **HR (95% CI)** | | ***p* value*** | | **HR (95% CI)** | | ***p* value*** | | **HR (95% CI)** | | ***p* value*** | |  |
| **Overall survival** |  |  | |  | |  | |  | |  | |  | |  |
| Treatment Factor | abTME | Reference | | **0.043** | | Reference | | **0.031** | | Reference | | **0.029** | |  |
|  | taTME | 2.66 (0.83-8.50) | |  | | 3.46 (0.96-12.50) | |  | | 3.03 (0.90-10.20) | |  | |  |
| Age |  | 1.04 (1.00-1.08) | | **0.013** | | 1.03 (0.99-1.07) | | 0.115 | | - | | - | |  |
| Sex | F | Reference | | 0.617 | | Reference | | 0.831 | | - | | - | |  |
|  | M | 1.19 (0.61-2.34) | |  | | 0.92 (0.42-1.99) | |  | | - | |  | |  |
| BMI |  | 0.98 (0.90-1.07) | | 0.660 | | 0.96 (0.89-1.03) | | 0.338 | | - | | - | |  |
| ASA classification | I/II | Reference 3 | | **<0.001** | | Reference | | **0.002** | | Reference | | **<0.001** | |  |
|  | III/IV | .55 (1.92-6.57) | |  | | 3.25 (1.62-6.52) | |  | | 3.37 (1.80-6.32) | |  | |  |
| Tumor height | < 6 cm | Reference | | 0.204 | | Reference | | 0.408 | | - | | **-** | |  |
|  | 6 to < 12 cm | 0.67 (0.35-1.28) | |  | | 0.65 (0.33-1.28) | |  | | - | | - | |  |
|  | 12 to 16 cm | 0.23 (0.04-1.51) | |  | | 0.35 (0.08-1.49) | |  | | - | | - | |  |
| Neoadjuvant therapy | No | Reference | | 0.943 | | Reference | | 0.790 | | - | | - | |  |
|  | Yes | 1.03 (0.52-2.03) | |  | | 1.11 (0.58-2.14) | |  | | - | |  | |  |
| Adjuvant therapy | No | Reference | | **0.023** | | Reference | | 0.052 | | Reference | | **0.032** | |  |
|  | Yes | 0.42 (0.21-0.84) | |  | | 0.45 (0.22-0.94) | |  | | 0.44 (0.22-0.87) | |  | |  |
|  |  |  | |  | |  | |  | |  | |  | |  |
| **Cancer-specific survival** |  |  | |  | |  | |  | |  | |  | |  |
| Treatment Factor | abTME | Reference | | 0.085 | | Reference | | **0.032** | | Reference | | 0.098 | |  |
|  | taTME | 3.21 (0.59-17.37) | |  | | 5.14 (0.92-28.74) | |  | | 3.12 (0.57-17.04) | |  | |  |
| Age |  | 1.02 (0.98-1.06) | | 0.411 | | 1.02 (0.98-1.06) | | 0.541 | | - | | - | |  |
| Sex | F | Reference | | 0.273 | | Reference | | 0.215 | | - | | - | |  |
|  | M | 0.61 (0.25-1.47) | |  | | 0.51 (0.17-1.49) | |  | | - | |  | |  |
| BMI |  | 0.92 (0.80-1.04) | | 0.136 | | 0.89 (0.79-1.02) | | 0.080 | | - | | - | |  |
| ASA classification | I/II | Reference | | **0.026** | | Reference | | **0.013** | | Reference | | **0.030** | |  |
|  | III/IV | 2.96 (1.30-6.77) | |  | | 3.96 (1.56-10.06) | |  | | 2.91 (1.26-6.71) | |  | |  |
| Tumor height | < 6 cm | Reference | | 0.266 | | Reference | | 0.469 | | - | | - | |  |
|  | 6 to < 12 cm | 0.46 (0.20-1.08) | |  | | 0.50 (0.18-1.40) | |  | | - | |  | |  |
|  | 12 to 16 cm | 0.39 (0.06-2.52) | |  | | 0.87 (0.13-5.77) | |  | | - | |  | |  |
| Neoadjuvant therapy | No | Reference | | 0.628 | | Reference | | 0.720 | | - | | - | |  |
|  | Yes | 1.29 (0.44-3.81) | |  | | 1.23 (0.43-3.54) | |  | | - | |  | |  |
| Adjuvant therapy | No | Reference | | 0.533 | | Reference | | 0.611 | | - | | - | |  |
|  | Yes | 0.72 (0.28-1.85) | |  | | 0.74 (0.26-2.09) | |  | | - | |  | |  |
|  |  |  | |  | |  | |  | |  | |  | |  |
| **Disease-free survival** |  |  | |  | |  | |  | |  | |  | |  |
| Treatment Factor | abTME | Reference | | 0.285 | | Reference | | 0.138 | | - | | - | |  |
|  | taTME | 1.47 (0.72-3.00) | |  | | 1.80 (0.83-3.92) | |  | | - | |  | |  |
| Age |  | 1.02 (1.00-1.05) | | 0.060 | | 1.01 (0.99-1.04) | | 0.278 | | - | | - | |  |
| Sex | F | Reference | | 0.447 | | Reference | | 0.302 | | - | | - | |  |
|  | M | 0.81 (0.49-1.35) | |  | | 0.74 (0.43-1.28) | |  | | - | |  | |  |
| BMI |  | 0.98 (0.92-1.05) | | 0.567 | | 0.97 (0.91-1.04) | | 0.422 | | - | | - | |  |
| ASA classification | I/II | Reference | | **0.011** | | Reference | | **0.044** | | Reference | | **0.011** | |  |
|  | III/IV | 2.05 (1.26-3.35) | |  | | 1.86 (1.08-3.20) | |  | | 2.05 (1.26-3.35) | |  | |  |
| Tumor height | < 6 cm | Reference | | 0.671 | | Reference | | 0.684 | | - | | - | |  |
|  | 6 to < 12 cm | 0.76 (0.43-1.36) | |  | | 0.78 (0.43-1.43) | |  | | - | |  | |  |
|  | 12 to 16 cm | 0.78 (0.29-2.13) | |  | | 1.07 (0.34-3.36) | |  | | - | |  | |  |
| Neoadjuvant therapy | No | Reference | | 0.988 | | Reference | | 0.993 | | - | | - | |  |
|  | Yes | 1.00 (0.58-1.71) | |  | | 1.00 (0.57-1.76) | |  | | - | |  | |  |
| Adjuvant therapy | No | Reference | | 0.103 | | Reference | | 0.182 | | - | | - | |  |
|  | Yes | 0.61 (0.36-1.06) | |  | | 0.65 (0.36-1.17) | |  | | - | |  | |  |
| HR: Hazard ratio; CI: confidence interval  A higher Hazard ratio expresses a higher risk for an unfavorable survival  *: Significant values are bold | |  |  | |  | |  | |  | |  | |  | |
